# Another Swim in the Extensive Pool of Zebrafish Research

**DOI:** 10.3390/biomedicines12030546

**Published:** 2024-02-29

**Authors:** James A. Marrs, Swapnalee Sarmah

**Affiliations:** Department of Biology, Indiana University Indianapolis, 723 West Michigan Street, Indianapolis, IN 46202, USA; swapnalee.sarmah@yahoo.com

## 1. Neural Systems

The zebrafish has gained utility in modeling biomedical phenomena for discovery research. Over 55,000 entries are found by searching the term “zebrafish” on PubMed. The creativity of the research community adds new approaches and targets for zebrafish models. Experiments using genome-wide methods have helped identify its mutations, transcription and chromatin structure. Genetic models are being applied to understand gene function and human disease. Experimental manipulations are powerful and reveal new mechanisms and biological knowledge stemming from zebrafish. This third volume of “Zebrafish Models for Development and Disease” adds new knowledge to the growing body of literature. An introduction highlighting a few of the papers in this Special Issue is presented below.

Traumatic brain injury is a widespread problem. Trauma in general is poorly understood and understudied. Growing attention is being paid to traumatic brain injury to support patients and to understand neural trauma [1,2]. David Hyde’s laboratory developed a consistent and reproducible model using adult zebrafish [3]. Their model produces a variety of injury phenotypes, from brain tissue disruptions to systemic effects, like edema and inflammation. The zebrafish is a regenerative model that allows investigators to evaluate these responses. Sonic hedgehog signaling was shown to regulate neural proliferation and regeneration responses following injury.

Alcohol consumption produces widespread societal problems. Progress is being made to identify its causation and treatment, including genetic interactions [4,5]. Animal models help us understand alcohol’s effects on the brain, and zebrafish have been used to great effect in Robert Gerlai’s laboratory. In their contribution to this Special Issue, adult zebrafish were treated with D1-dopamine-receptor antagonists and alcohol to determine whether these effects interact [6]. Experiments compared inbred AB and heterogenous SFWT genetic strains. They found synergy between D1-dopamine-receptor antagonists and alcohol for shoaling behavior but found additive effects on exploratory behavior. They next tested the effects of D1-dopamine-receptor antagonists and alcohol treatments on neurotransmitter levels and found interactions between dopamine and DOPAC (metabolite of dopamine) levels. Only alcohol affected the serotonin levels, showing specificity in their model. Behavior paradigms are needed to extend zebrafish models’ utility in neuroscience, and the groundwork in this study is important and necessary.

## 2. Tumor Biology

Zebrafish are versatile for producing animal models for various conditions, including tumor progression [7]. Sara Rezzola and colleagues developed a zebrafish platform to evaluate drug efficacy on human and mouse uveal melanoma cells [5]. They transplanted uveal melanoma tumor xenografts into the zebrafish eye near the developing choroid vasculature. These cells grew and invaded the eye tissues. Using a luciferase-expressing cell line, they quantified the tumor response to chemotherapeutic drugs. This is an exciting addition to the assay arsenal.

Inflammation contributes to tumor progression; this was studied in the zebrafish in Zhiyuan Gong’s laboratory using a transgenic *krasV12-*expressing oncogene in the intestine and using lipopolysaccharide or dextran sulfate sodium inflammatory compounds as treatment [7]. Treatment with the inflammatory compounds in the transgenic *krasV12*-expressing fish increased neutrophils and macrophages in the intestine. The synergy of the oncogene and inflammation produced more hyperplasia and tumorogenesis, including specific cellular proliferation, apoptosis and signaling responses. Tumor progression changed the cellular composition and morphology in the intestine. The authors plan to use this model to investigate tumor initiation mechanisms and test antitumor drugs.

## 3. Liver Biology

Exploiting zebrafish as models for liver biology represents an opportunity to analyze, validate and consolidate our understanding derived from other models, like rodents [8]. Zebrafish are outstanding developmental models, and the study conducted in Zongbin Cui’s laboratory used the fish system to characterize the transcriptome changes during liver development [8]. Hepatocytes were sorted using flow cytometry at three stages. The authors identified genes whose transcription changed over these times. Gene ontogeny (GO) was used to categorize the types of activities that changed during development. Initial changes (60 to 72 h postfertilization; hpf) were seen in the cell cycle, DNA replication, DNA repair, RNA processing and transcription regulation. Later (72 to 96 hpf), the Kyoto Encyclopedia of Genes and Genomes (KEGG) was used to categorize the changes that occurred in the hepatocytes, including changes in the cell cycle, RNA degradation, ubiquitin-mediated proteolysis, signaling pathways, basal transcription factors and glycan degradation. These pathways included similar activities as those seen at the earlier stages. In addition, the metabolic pathways were upregulated, including nucleic acid bases, energy carriers, amino acids, ABC transporters and p53, which participates in many pathways. This study will provide a guide for future studies of liver development, stem cell biology and liver disease processes.

## 4. Kidney Biology

The kidney is an organ that highlights simplicity in the zebrafish system; it only has one fused glomerulus and two nephrons in the embryo [9], but still has the powers of genetics and regenerative capacity found in zebrafish [10]. Genetic defects, like polycystic kidney disease, are modeled in zebrafish [11], as are other ciliopathies [12]. Indeed, zebrafish are useful models to study kidney development and disease [13,14].

In Dr. Rebecca Wingert’s laboratory, Drummond et al. identified the *osr1* gene mutation that affects kidney development [15]. This gene normally promotes the podocyte lineage, which are the cells forming the glomerulus filter. Osr1 is a zinc finger transcription factor, and the authors show that Osr1 promotes the expression of the paracrine signaling ligand Wnt2ba. Indeed, they show that Wnt2ba expression can partially rescue *osr1* mutant fish, allowing for renal podocyte development. They illustrate that the Osr1 and Hand2 transcription factors antagonize each other, regulating the podocyte progenitor pathway. This is an exceptional study showing the power of zebrafish models to dissect complex developmental biology pathways.

## 5. Conclusions

Overall, this Special Issue illustrates the spectrum of ways that zebrafish can be applied in biomedical research. New and creative approaches continue to uncover knowledge about development and disease. New technologies, particularly genome-wide methods, are helping us to understand biological and disease processes. The future is bright, and there are extraordinary possibilities for growth in the zebrafish research ecosystem.

## Data Availability

Not applicable.
